# Comprehensive evaluation of growth performance and meat characteristics of a fattening system combining grazing with feeding rice whole‐crop silage in Japanese Black steers

**DOI:** 10.1111/asj.13176

**Published:** 2019-01-20

**Authors:** Masahiro Shibata, Yasuko Hikino, Mai Imanari, Kazunori Matsumoto

**Affiliations:** ^1^ Faculty of Applied Life Science Nippon Veterinary and Life Science University Musashino Tokyo Japan; ^2^ Livestock Production and Wildlife Management Research Division NARO Western Region Agricultural Research Center Oda Shimane Japan; ^3^ Livestock and Forage Research Division NARO Tohoku Agricultural Research Center Morioka Iwate Japan

**Keywords:** beef productivity, carcass and meat characteristics, gene expression, grazing, rice whole‐crop silage

## Abstract

We investigated the effect of a system for fattening steers combining grazing with feeding rice whole‐crop silage (rWCS) on growth performance, meat characteristics, and the expression of genes involved in skeletal muscle growth. Steers were randomly assigned to grazing or concentrate‐fed groups (CT). The grazing group (GZ) was fed rWCS after grazing until 16 months of age. The final body weight was the same in the two groups, but the dressed weight was lower in the GZ than in the CT. The beef color standard was higher in the GZ than in the CT. Although beef marbling did not differ between the two groups, there was less intramuscular fat and subcutaneous fat in the GZ than in the CT. The α‐tocopherol and β‐carotene contents in the muscle were higher in the GZ than in the CT. The GZ showed a lower daily gain (DG) during the grazing period, which may have resulted from decelerating skeletal muscle growth caused by the increased expression of genes encoding myostatin and atrogin‐1. However, the DG and feed efficiency of the GZ increased after grazing. The two groups exhibited a similar level of beef productivity.

## 1 INTRODUCTION

The annual report on food, agriculture, and rural areas in Japan has suggested that switching from imported feed to domestic feed is important for enhancing the livestock farming infrastructure because the feed self‐sufficiency rate for livestock production in Japan in 2015 was very low (28%, MAFF, 2018). It is important to carefully evaluate beef cattle fattening systems using domestically produced feed independent of imported grain feed. In Japan, beef cattle are generally finished indoors on a concentrate‐based diet throughout the fattening period until slaughter. However, several studies on the different methods of fattening beef cattle have investigated the effects of supplying roughage, such as rice whole‐crop silage (rWCS) (Shibata, Hikino, Imanari, Matsumoto, & Yamamoto, 2016; Takahira et al., 2011), grass hay (Muramoto, Aikawa, Shibata, & Nakanishi, 2002; Shibata, Hikino, & Matsumoto, 2019), and grazing (Descalzo et al., 2005; Muramoto, Higashiyama, & Kondo, 2005; Pavan & Duckett, 2008), instead of concentrate diets. These previous reports showed that beef cattle can be fattened by feeding roughage or by grazing without necessarily depending on concentrate‐based diet, thereby affecting the growth performance, carcass characteristics, and meat characteristics of the steers. Furthermore, the use of grazing is expected to reduce the operational costs because the steers do not need barns. Although these previous reports covered fattening beef cattle by grazing or rWCS feeding, there have been no reports on fattening beef cattle by combining grazing with rWCS feeding, independent of a concentrate‐based diet.

Changes in gene expression may reflect biological responses in the body during fattening. Although gene expression cannot be used for monitoring the growth of animals on the farm, monitoring gene expression can provide useful information and scientific evidence for research institutes to develop novel fattening systems. Myostatin is a negative regulator of skeletal muscle growth, and functions in both the hyperplasia and hypertrophy of skeletal muscle (McPherron, Lawler, & Lee, 1997). Proteolysis by the ubiquitin–proteasome system occurs by selective binding of E3 ubiquitin ligases to the target protein. Atrogin‐1 (muscle atrophy F‐box) and muscle RING Finger 1 are E3 ubiquitin ligases found exclusively in skeletal muscle, and are the dominant mediators of skeletal muscle wasting (Bodine et al., 2001; Gomes, Lecker, Jagoe, Navon, & Goldberg, 2001). We suggest that myostatin and atrogin‐1 can provide valuable information on skeletal muscle growth during the fattening period.

In this study, we aim to investigate the influence of a system of fattening steers which combines grazing with feeding rWCS, independent of concentrate diets, on the growth performance, meat characteristics, and the expression of genes involved in skeletal muscle growth. Grazing steers have no restriction on their exercise, whereas steers fattened conventionally are restricted in their exercise by living in a barn. Although the living environments are different, this study aims to evaluate and compare the two fattening systems, using steers fed concentrate‐based diets as the control. We hypothesize that growth performance might temporarily be inferior due to grazing but would recover after subsequent feeding with rWCS. Therefore, we suggest that the final growth performance of the two groups of steers will be similar.

## 2 MATERIALS AND METHODS

All experimental procedures and the management of steers in this study were performed in accordance with the Animal Experimental Guidelines of NARO Western Region Agricultural Research Center (NARO/WARC) established by its Animal Experimental Committee, which approved the study (certification number—13‐CHIKUSANSOUCHI‐01).

### 2.1 Animal management

Ten Japanese Black steers, aged 10 months (10.7 ± 0.12 months), bred at the NARO/WARC, were selected randomly and divided into two groups: a grazing group (GZ) and a concentrate‐fed group (CT). The GZ group (*n *=* *5) was grazed rotationally on Italian ryegrass pasture from 10 to 16 months of age, then each steer was reared individually in a tie‐stall barn and fed on a diet of rWCS and concentrate (flaked corn, flaked barley, wheat bran, and soybean meal; 82.3% total digestible nutrients [TDN] and 12.2% crude protein [CP] on a dry matter basis) from 16 to 28 months of age. During the grazing period, the grazing steers were gathered at a feeding station every day and fed 200 g concentrate, and their body conditions observed. After the grazing period, the GZ group was fed an experimental diet consisting of rWCS (57% TDN and 6.0% CP on a dry matter basis) ad libitum with restricted feeding of the concentrate diet in a tie‐stall barn until 28 months of age. From 16 to 22 months of age, the GZ group was fed the concentrate diet at 4–8 kg/day, and from 22 to 28 months of age at 2.5–3.5 kg/day. During the fattening period, the experimental diet of the GZ group was supplemented with soybean meal (81% TDN and 50% CP on a dry matter basis) to make up for the reduction in CP caused by the decreased amount of the concentrate diet. The steers in the CT group (*n *=* *5) were managed individually in a tie‐stall barn and fed the concentrate diet ad libitum and Italian ryegrass hay (49% TDN and 5.3% CP on a dry matter basis) at 1.5–2.0 kg/day throughout the whole‐fattening period from 10 to 28 months of age.

The body weight (BW) of each steer was recorded every 1–2 weeks. Food intake was measured daily except during the grazing period. The grass mass was measured periodically during the grazing period and its chemical composition was analyzed by the Agricultural Chemistry Research Institute (Hokkaido, Japan) (Table 1). The rice used in the feed was of the Tachisuzuka cultivar, which was harvested at the yellow ripening stage. Its chemical composition was analyzed by the Agricultural Chemistry Research Institute (Table 2). The steers were slaughtered at 28 months of age, and the skeletal muscle tissue was collected to analyze the meat characteristics. The TDN intake was calculated from the feed intake and the estimated TDN in each diet.

**Table 1:** Variation in the mass and nutritional composition of grass during the period of grazing

Period (month)^a^	Grass mass (kg of DM/ha)	TDN (% of DM)	CP (% of DM)	ADF (% of DM)	NDF (% of DM)
10–11	1,759	76.0	21.6	18.3	41.2
11–12	2,093	71.3	17.0	18.6	39.8
12–13	3,705	72.2	20.9	20.5	49.0
13–14	4,090	72.8	12.7	25.0	49.2
14–15	2,715	72.7	12.8	25.9	48.9
15–16	4,444	70.9	8.8	36.0	62.0

*Notes*.

TDN: total digestible nutrients; CP: crude protein; ADF: acid detergent fiber; NDF: neutral detergent fiber.

^a^Period: age of steers in months, 10–13 months; winter, 13–16 months; spring.

**Table 2:** Nutritional composition of rice whole‐crop silage

	TDN (% of DM)	CP (% of DM)	ADF (% of DM)	NDF (% of DM)
Rice whole‐crop silage	57.2	6.01	33.6	57.2

*Note*.

TDN: total digestible nutrients; CP: crude protein; ADF: acid detergent fiber; NDF: neutral detergent fiber.

### 2.2 Sample collection

The samples of skeletal muscle tissues from the *M*. *longissimus lumborum* (LL) and *M. semitendinosus* (ST) of both groups were obtained by biopsy at 10, 13, 16, 19, 22, and 28 months of age to analyze gene expression. The biopsies at 10 and 16 months of age were carried out before grazing and just before the end of grazing, respectively. The biopsy procedure was as follows: the animal was locally anesthetized by an intramuscular injection of xylazine (Bayer, Tokyo, Japan) and a subcutaneous injection of lidocaine (AstraZeneca, Osaka, Japan). An incision was subsequently made in the skin overlying the LL and ST muscles (Shibata et al., 2006). All biopsy samples were rapidly frozen in liquid nitrogen and stored at −80°C until the RNA was extracted.

### 2.3 Carcass evaluation and sample preparation

Carcasses were kept in a refrigerator at 0°C for 24 hr before evaluation. In accordance with the Japanese New Beef Carcass Grading Standards (JMGA, 1988), carcasses were evaluated according to dressing percentage, beef marbling standard (BMS) number, beef fat color standard (BFS), beef color standard (BCS), and by measuring rib eye area, rib thickness, and subcutaneous fat thickness of the section between the sixth and seventh ribs. The skeletal muscle tissue samples of the LL and ST muscles were obtained from the carcasses to analyze several meat characteristics such as drip loss, cooking loss, and Warner–Bratzler (WB) shear force. The LL and ST muscles were processed into 2.5‐cm thick steaks, vacuum‐packed, stored in a refrigerator at 2°C for 2 and 30 days after slaughter, respectively, and then frozen at −80°C until analysis.

### 2.4 Meat characteristics

The muscle tissues were minced before their nutrient contents were determined. CP was calculated by quantitative analysis of nitrogen using the Kjeldahl method with copper sulfate and potassium sulfate as catalysts (AOAC, 1990). Lipids were extracted with diethyl ether for 16 hr using a Soxhlet extractor (AOAC, 1990). To analyze the fatty acid composition in the muscle tissue, the extracted lipids were converted to fatty acid methyl esters using a boron trifluoride/methanol complex in methanol solution then analyzed using gas chromatography (AOCS, 2005).

The frozen steaks were thawed for 24 hr at 4°C then carefully blotted dry with paper tissues. The drip loss was calculated from the weight difference between before and after storage (Muramoto et al., 2005). After the drip loss measurement, the samples were broiled on electric grills to an internal temperature of 70°C then wrapped in plastic to prevent desiccation and stored at 4°C for approximately 12 hr (Montgomery et al., 2001; Realini et al., 2005). Cooking loss was calculated from the difference between the weights before and after cooking (Montgomery et al., 2001; Muramoto et al., 2005). Six cores (1.3 cm in diameter) were removed from each steak parallel to the longitudinal orientation of the muscle fibers (Montgomery et al., 2001; Realini et al., 2005). All cores were sheared using a WB shear force machine, and the peak shear force was recorded. In this study, only the drip loss and cooking loss were used as indices of water‐holding capacity.

### 2.5 RNA isolation, cDNA synthesis, and measurement of gene expression

The total RNA was extracted from the muscle tissue samples using Trizol reagent (Invitrogen Corp., Carlsbad, CA) according to the manufacturer's protocol. First‐strand complementary DNA (cDNA) was synthesized from 3 μg total RNA using SuperScript II RNase H^−^ reverse transcriptase with oligo dT primer (Invitrogen Corp.).

After reverse transcription, the transcript levels of genes encoding myostatin and atrogin‐1 were determined by real‐time PCR using an ABI 7500 detection system (Applied Biosystems, Foster City, CA, USA). The first‐strand cDNA was diluted with deionized water then amplified using TaqMan gene expression master mix (Applied Biosystems) with a gene‐specific TaqMan probe and primers (Table 3). The real‐time PCR thermal cycling program was as follows: 2 min at 50°C, 10 min at 95°C, 50 cycles of 15 s at 95°C and 1 min at 60°C. The housekeeping gene *GAPDH*, encoding glyceraldehyde‐3‐phosphate dehydrogenase, was used as a normalization control because proteome analysis has shown that there was no change in the expression of intramuscular *GAPDH* in grazed cattle compared with concentrate‐fed cattle (Shibata et al., 2009). The TaqMan probe and primers were designed using Primer Express (Applied Biosystems).

**Table 3:** Sequences of real‐time PCR primers used in this study^a^

Gene^b^	GenBank accession no.	Forward primer (5′–3′)	Reverse primer (5′–3′)	Product size, bp
*Myostatin*	AB076403	GGCCATGATCTTGCTGTAACCT	GCATCGAGATTCTGTGGAGTG	144
*Atrogin I*	NM1046155	GTTGTACGGCTGTTGGAACTGA	GCAGGAGCTCCCTTATTAGTC	148
*GAPDH*	U85042	TGACCCCTTCATTGACCTTCA	ACCCCAGTGGACTCCACTACAT	201

^a^All sequence data are bovine sequences. ^b^GAPDH, glyceraldehyde‐3‐phosphate dehydrogenase.

### 2.6 Statistical analyses

All data are presented as means ± *SEM*. The data from the GZ and CT groups were analyzed using one‐way analysis of variance (ANOVA) and the post hoc Fisher's probability least significant difference test. Serial changes in gene expression data were analyzed using repeated measured ANOVA. If ANOVA revealed significant differences, then each dataset was assessed using the post hoc Scheffé test. A *p*‐value of <0.05 was considered to be statistically significant.

## 3 RESULTS

### 3.1 Growth performance and carcass characteristics

Table 4 shows the effects of grazing and feeding rWCS on the growth performance and feed intake of steers. The BW at 16 months of age, after grazing, tended to be lower for the GZ group than for the CT group (*p *=* *0.088), but the middle and final BW were not significantly different between the two groups (*p *>* *0.05). The daily gain (DG) during the grazing period (10–16 months) was significantly lower in the GZ group than in the CT group (*p *<* *0.05). The DG during the rWCS feeding Period 1 (16–22 months) was significantly greater in the GZ group than in the CT group (*p *<* *0.01), whereas the DG in the rWCS feeding Period 2 (22–28 months) was significantly lower in the GZ group than in the CT group (*p *<* *0.05). The total TDN intake and feed efficiency during the rWCS feeding Period 1 was significantly higher in the GZ group than in the CT group (*p *<* *0.01). However, the TDN intake and feed efficiency during the rWCS feeding Period 2 was significantly lower in the GZ group than in the CT group (*p *<* *0.01).

**Table 4:** Growth performance and feed intake of steers (GZ, grazed group; CT, concentrate‐fed group)

	GZ	CT
Body weight (BW), kg		
Before grazing (10 month)	331 ± 7.97	328 ± 11.4
After grazing (16 month)	469 ± 14.1#	507 ± 13.5
Middle (22 month)	661 ± 6.43	647 ± 10.6
Final (28 month)	705 ± 9.14	728 ± 8.66
Daily gain, kg/day		
Grazing period (10–16 months)	0.76 ± 0.04*	0.99 ± 0.05
WCS feeding period (16–28 months)	0.67 ± 0.02	0.64 ± 0.02
WCS feeding Period 1 (16–22 months)	0.98 ± 0.05*	0.71 ± 0.02
WCS feeding Period 2 (22–28 months)	0.28 ± 0.03*	0.56 ± 0.05
TDN intake, kg/day		
Grazing period		6.20 ± 0.15
WCS feeding period	6.14 ± 0.74	6.01 ± 0.19
WCS feeding Period 1	6.98 ± 0.08*	6.05 ± 0.17
WCS feeding Period 2	5.09 ± 0.15*	5.99 ± 0.25
Feed efficiency, BW gain/TDN intake		
Grazing period		0.159 ± 0.005
WCS feeding period	0.109 ± 0.003	0.107 ± 0.002
WCS feeding Period 1	0.140 ± 0.006*	0.118 ± 0.002
WCS feeding Period 2	0.055 ± 0.005*	0.093 ± 0.007

*Notes*.

Values are *M*s ± *SEM*.

TDN: total digestible nutrients.

*p <* *0.05, ^#^ *p *<* *0.10.

Table 5 shows the effects of grazing and feeding rWCS on the carcass characteristics of the steers. Although there was no significant difference in the final BW between the two groups (*p *>* *0.05), the dressed weight was significantly lower for the GZ group than for the CT group (*p *<* *0.01). The rib eye area of the GZ group tended to be smaller than that of the CT group (*p *=* *0.080), which is a positive evaluation under the Japanese New Beef Carcass Grading Standards. In contrast, the subcutaneous fat thickness was significantly smaller in the GZ group than in the CT group, which is a negative evaluation under these same standards (*p *<* *0.05). The BCS and BFS were significantly higher in the GZ group than in the CT group (*p *<* *0.05). However, there were no significant differences in the dressing percentage, rib thickness, and BMS between the two groups, indicating that there was no difference in beef quality.

**Table 5:** Carcass characteristics of steers (GZ, grazed group; CT, concentrate‐fed group)

	GZ	CT
Dressed weight, kg	423 ± 6.13*	459 ± 4.94
Dressing percentage, %	72.2 ± 0.40	71.8 ± 0.12
Rib eye area, cm^2^	46.0 ± 2.05#	51.2 ± 1.59
Rib thickness, cm	7.16 ± 0.30	7.32 ± 0.14
Subcutaneous fat thickness, cm	2.98 ± 0.25*	3.86 ± 0.20
Beef marbling standard, No.	3.4 ± 0.25	3.2 ± 0.20
Beef color standard, No.	4.8 ± 0.20*	3.8 ± 0.20
Beef fat color standard, No.	5.8 ± 0.20*	3.0 ± 0.00

*Notes*.

Values are *M*s ± *SEM*.

*p <* *0.05, ^#^ *p *<* *0.10.

### 3.2 Meat characteristics

Table 6 shows the effects of grazing and feeding rWCS on the nutrient contents, water‐holding capacity, and WB shear force of the steer skeletal muscle. The extractable lipid content in the LL muscle was significantly lower in the GZ group than in the CT group (*p *<* *0.05). A similar trend was observed in the extractable lipid content of the ST muscle, but the difference between the two groups was not significant (*p *=* *0.077). The CP content in the LL muscle tended to be higher in the GZ group than in the CT group, but the difference was not significant (*p *=* *0.058). The LL and ST muscles of the GZ group tended to have higher moisture contents than those of the CT group, but these differences were not significant (*p *=* *0.052 and 0.065, respectively). The β‐carotene and α‐tocopherol contents in both muscles were significantly greater in the GZ group than in the CT group (*p *<* *0.01). The polyunsaturated fatty acid (PUFA) content in the LL muscle was significantly lower in the GZ group than in the CT group (*p *<* *0.01). The n‐6/n‐3 ratios in both muscles were significantly lower in the GZ group than in the CT group (*p *<* *0.05). In terms of water‐holding capacity, the drip loss and cooking loss from both muscles did not differ between the two groups. The WB shear force of the LL muscle at 30 days after slaughter tended to be lower in the GZ group than in the CT group, but this difference was not significant (*p *=* *0.073).

**Table 6:** Nutrient contents, vitamin contents, water‐holding capacity, and Warner–Bratzler shear force in the *M. longissimus lumborum* (LL) and *M*. *semitendinosus* (ST) muscles of steers (GZ, grazed group; CT, concentrate‐fed group)

	LL		ST	
	GZ	CT	GZ	CT
Crude protein, %	18.2 ± 0.48#	16.6 ± 0.58	20.8 ± 0.20	19.8 ± 0.55
Extract lipid, %	21.7 ± 1.74*	27.2 ± 1.62	6.38 ± 0.55#	9.86 ± 1.63
Moisture, %	59.5 ± 1.33#	55.4 ± 1.22	71.8 ± 0.40#	69.4 ± 1.07
Vitamin				
Retinol, μg/100 g	26.2 ± 3.01	21.9 ± 0.87	7.00 ± 0.71	8.52 ± 1.75
β‐Carotene, μg/100 g	21.0 ± 3.27*	3.20 ± 1.10^a^	8.80 ± 0.49*	1.05 ± 0.05^a^
α‐Tocopherol, mg/kg	17.2 ± 1.02*	3.35 ± 0.79	12.1 ± 0.48*	2.00 ± 0.24
Fatty acid composition, %				
SFA	40.7 ± 1.35	40.9 ± 1.73	37.0 ± 1.66	37.6 ± 1.41
MUFA	55.0 ± 1.42	55.9 ± 1.48	56.0 ± 1.69	58.0 ± 1.41
PUFA	1.40 ± 0.15*	2.22 ± 0.18	3.04 ± 0.20	3.18 ± 0.32
n‐6/n‐3	7.17 ± 0.17*	10.8 ± 1.17^a^	9.26 ± 1.27*	14.7 ± 1.59^a^
Water‐holding capacity				
Drip loss, %	2.24 ± 0.12	2.38 ± 0.33	5.49 ± 0.18	6.49 ± 0.80
Cooking loss, %	36.6 ± 0.90	37.1 ± 2.06	39.8 ± 0.54	38.5 ± 1.03
Shear force, kg				
2 day	2.02 ± 0.18	1.84 ± 0.17	4.40 ± 0.39	3.63 ± 0.33
30 day	1.05 ± 0.04#	1.28 ± 0.10	2.48 ± 0.14	2.38 ± 0.15

*Notes*.

Values are *M*s ± *SEM*.

SFA: sum of saturated fatty acids (14:0, 15:0, 16:0, 17:0, 18:0); MUFA: sum of monounsaturated fatty acids (14:1, 16:1, 17:1 18:1, 20:1); PUFA: sum of polyunsaturated fatty acids (18:2, 18:3, 20:3, 20:4); n‐6/n‐3: (18:2n‐6 + 20:3n‐6 + 20:4n‐6)/(18:3n‐3 + 22:5n‐3).

^a^n‐3 PUFAs were not detected in two samples of both muscles in CT group.

*p <* *0.05, ^#^ *p *<* *0.10.

### 3.3 Gene expression in skeletal muscle

**Figure 1:**
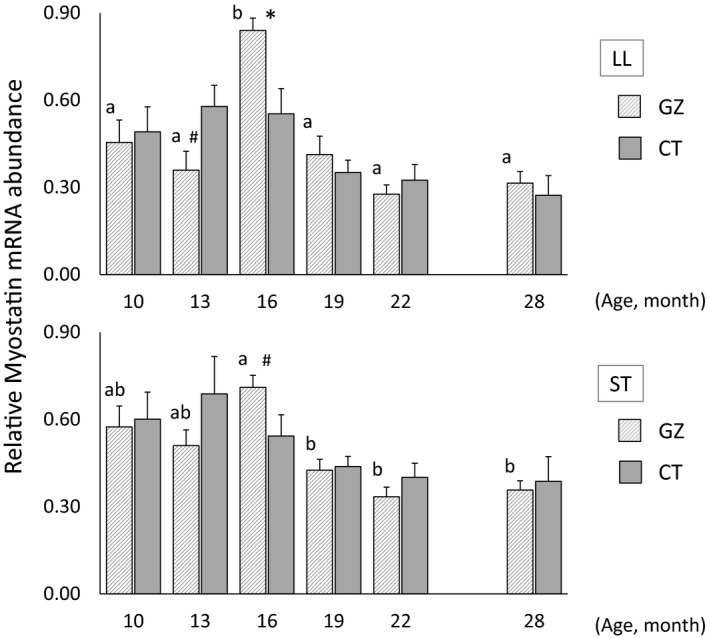
Developmental changes in *myostatin* gene expression in the *longissimus lumborum* (LL) and *semitendinosus* (ST) muscles of Japanese Black steers assessed by real‐time PCR. Transcript level of *myostatin* was normalized to *GAPDH*. Data are *M* ± *SEM* at each point. *p < 0.05, compared with the same age. ^#^ *p *< 0.10, compared with the same age. Values for different ages sharing a common letter are not significantly different (*p* > 0.05). GZ: grazing group; CT: concentrate‐fed group

Figure 1 shows the changes in the transcript levels of *myostatin* in the skeletal muscle of steers grazed and fed rWCS. The *myostatin* transcript levels in the LL muscle at the end of the grazing period (16 months of age) were significantly higher in the GZ group than in the CT group (*p *<* *0.05). In contrast, the transcript level of *myostatin* in the LL muscle at 13 months of age tended to be lower in the GZ group than in the CT group, but this difference was not significant (*p *=* *0.055). The highest transcript level of *myostatin* in the LL muscle was in the GZ group at 16 months of age (*p *<* *0.01), and thereafter the transcript level was low until 28 months of age. In the ST muscle, the *myostatin* transcript levels tended to be higher in the GZ group than in the CT group at the end of the grazing period, but this difference was not significant (*p *=* *0.082). The transcript level of *myostatin* in the ST muscle of the GZ group was significantly higher at 16 months of age than during the rWCS feeding period (16–28 months of age) (*p *<* *0.05).

**Figure 2:**
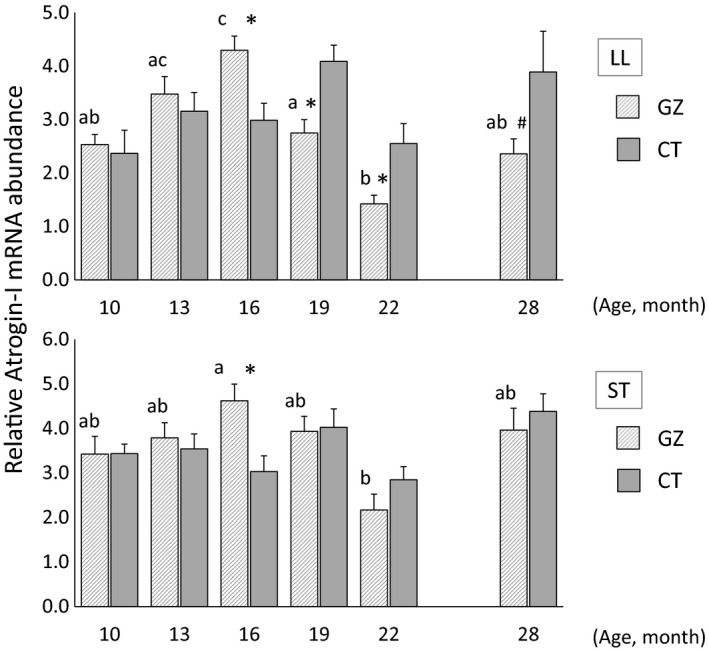
Developmental changes in *atrogin‐1* gene expression in the *longissimus lumborum* (LL) and *semitendinosus* (ST) muscles of Japanese Black steers assessed by real‐time PCR. Transcript level of *atrogin‐1* was normalized to *GAPDH*. Data are *M* ± *SEM* at each point. *p < 0.05, compared with the same age. ^#^ *p *< 0.10, compared with the same age. Values for different ages sharing a common letter are not significantly different (*p *> 0.05). GZ: grazing group; CT: concentrate‐fed group

The changes in the *atrogin‐1* transcript levels in steer skeletal muscles are shown in Figure 2. The *atrogin‐1* transcript levels in the LL and ST muscles at the end of the grazing period (16 months of age) were significantly higher in the GZ group than in the CT group (*p *<* *0.05). In contrast, the *atrogin‐1* transcript levels in the LL muscle at 19 and 22 months of age were significantly lower in the GZ group than in the CT group (*p *<* *0.05). The transcript levels of this gene in the LL muscle tended to be lower in the GZ group than in the CT group at 28 months of age, but this difference was not significant (*p *=* *0.096). In terms of changes during the fattening stage, the *atrogin‐1* transcript levels in the LL muscle of the GZ group peaked at 16 months of age (*p *<* *0.05), and thereafter remained at low levels up to 28 months of age. The *atrogin‐1* transcript level in the ST muscle of the GZ group was significantly higher at 16 months of age than at 22 months of age (*p *<* *0.01).

## 4 DISCUSSION

### 4.1 Growth performance and gene expression

This study aimed to investigate the growth performance, carcass and meat characteristics, and expression patterns of genes involved in muscle growth in fattening system for steers combining grazing with feeding rWCS. Steers grazed on tall fescue pasture with or without a supplement diet have been reported to exhibit a lower DG, a smaller *longissimus* muscle (LM) area, and reduced carcass weight compared with those fed a concentrate‐based diet (Pavan & Duckett, 2008). Pasture‐fed steers had a lower DG than that of grain‐fed steers, so they were allowed to continue grazing until they reached slaughter weight (Descalzo et al., 2005). Therefore, grazing may be less effective than other diets for beef production because of a decrease in the DG and low productivity. However, feeding a rWCS‐based diet to steers maintained the same productivity level of beef as feeding a concentrate‐based diet (Shibata et al., 2016). Thus, we can suggest that rWCS could be an effective alternative to a concentrate‐based diet. Although the DG during the grazing period was lower in the GZ group than in the CT group, an increase in the DG after grazing resulted from improved TDN intake and feed efficiency. These results suggest that changes in the rearing environment affected the growth performance of the GZ group. Steers have been shown to exhibit compensatory growth after switching from low‐nutrient to high‐nutrient conditions (Carstens, Johnson, Ellenburger, & Tatum, 1991; Horton & Holmes, 1978). Although the nutritional intake of steers during the grazing period was not clear, compensatory growth may have occurred during the rWCS feeding Period 1 because of the higher DG, TDN intake, and feed efficiency of the GZ group during that period. However, the compensatory growth in the GZ group was not maintained until the end of fattening. This may be the result of a reduction in TDN intake caused by the restricted feeding of the concentrate diet during the late fattening period. Further studies are needed to investigate the full exploitation of compensatory growth in this rearing system.

Analyses of gene expression can provide information about biological reactions in the body during the fattening periods. Studies on myostatin‐null mice have shown that myostatin functions as a specific negative regulator of skeletal muscle growth (McPherron et al., 1997). Natural mutations of the gene encoding myostatin are known to be responsible for the double‐muscle phenotype of several breeds of steers (Grobet et al., 1997; Kambadur, Sharma, Smith, & Bass, 1997; McPherron & Lee, 1997). Atrogin‐1 is an E3 ubiquitin ligase found exclusively in skeletal muscle, and is the dominant mediator of skeletal muscle wasting (Bodine et al., 2001; Gomes et al., 2001). An increase in the expression of genes involved in the ubiquitin–proteasome proteolytic pathway is associated with skeletal muscle wasting (Lecker et al., 2004; Wing, Haas, & Goldberg, 1995). Skeletal muscle growth in rWCS‐fed steers may be activated in the late fattening period because of the decreased expression of *myostatin* (Shibata et al., 2016). The *myostatin* transcript levels in the GZ group suggest that the skeletal muscle growth of the steers may have been suppressed during the late grazing period. In addition, the transcript levels of *atrogin‐1* in the GZ group suggest that skeletal muscle proteolysis in the steers during the late grazing period was enhanced by increasing the activity of the ubiquitin–proteasome system. Thus, the decrease in the DG of grazed steers may have resulted from the deceleration of skeletal muscle growth by the increased expression of *myostatin* and *atrogin‐1* in the late grazing period. In contrast, *myostatin* and *atrogin‐1* transcript levels were low in the GZ group after grazing. These results suggest that, in the GZ group, skeletal muscle growth may be activated in the rWCS feeding period because of the decreased expression of *myostatin* and *atrogin‐1* during that period. Compensatory growth has been reported to be associated with the expression of *myostatin* and *MyHC* in the skeletal muscle of grass hay‐fed steers (Shibata et al., 2011). The expression patterns of the *myostatin* and *atrogin‐1* genes suggest that compensatory growth may have occurred in the GZ group during the rWCS feeding period, and that the expression of these genes support the findings on the levels of DG, TDN intake and feed efficiency in the GZ group.

### 4.2 Carcass characteristics

The final BW did not differ between the two groups but the dressed weight was lower in the GZ group than in the CT group. Feeding a grass hay‐based diet to steers did not alter the final BW, but resulted in a lower dressed weight than that of the concentrate‐fed steers (Shibata et al., 2019). Furthermore, the final BW and dressed carcass weight were lower in a large amount of grass hay‐fed steers than in concentrate‐fed steers (Shibata et al., 2012). Steers grazed on tall fescue pasture had a smaller LM area, a thinner subcutaneous fat thickness, and a lower marbling score compared with steers fed a concentrate‐based diet (Pavan & Duckett, 2008). Cows grazed on native pasture had a smaller LM area and lower carcass weight than cows fed on concentrate but the subcutaneous fat thickness did not differ between the two groups (Neill et al., 2008). Steers fed a pasture‐forage diet had a thinner subcutaneous fat thickness, smaller rib eye area, and lower marbling score and carcass weight than concentrate‐fed steers (Bjorklund, Heins, DiCostanzo, & Chester‐Jones, 2014). Compared with a concentrate‐based diet, a diet containing a large amount of grass hay during the early fattening period led to a thinner subcutaneous fat thickness and rib thickness in steers (Shibata et al., 2019). These results indicated that grazing or feeding roughage to steers leads to a decreased LM area and dressed carcass weight and a thinner subcutaneous fat thickness compared with steers fed a concentrate‐based diet. The results of this study agree with previous reports in which steers were grazed or fed on roughage.

### 4.3 Meat characteristics

In general, the color of the lean meat from grazed steers was dark because of the higher myoglobin content with an increase in the proportion of slow‐twitch fibers in the muscle. Pasture‐finished steers exhibited increased contents of slow‐twitch fibers in the muscle (Shibata, Matsumoto, Hikino, & Yamamoto, 2014). Grazed steers exhibited increased myoglobin contents and a higher proportion of slow‐twitch fibers in the muscle compared with concentrate‐fed steers (Shibata et al., 2009). In addition, pasture‐forage‐fed steers were found to have a darker lean meat color and higher myoglobin content than grain‐fed steers (Bidner et al., 1986). The higher BCS in the GZ group suggest that grazing possibly led to an increase in myoglobin in the slow‐twitch muscle fibers.

The α‐tocopherol and β‐carotene contents in muscle have been shown to be higher in pasture‐fed steers than in grain‐fed steers (Descalzo et al., 2005) and in concentrate‐fed steers (Muramoto et al., 2005). Similarly, the α‐tocopherol content in muscle was higher in rWCS‐fed steers than in concentrate‐fed steers (Shibata et al., 2016). These results are consistent with the results of this study.

n‐3 PUFAs reduce the risk of cardiovascular disease (Calder, 2004). It is recommended that the value of the n‐6/n‐3 ratio, used as an index of human health, should be less than four (Department of Health, 1994). The n‐6/n‐3 ratio can be lowered by fattening steers on a roughage diet such as pasture (Descalzo et al., 2005; Muramoto et al., 2005; Varela et al., 2004), grass silage (Warren et al., 2008), or rWCS (Shibata et al., 2016). Although the n‐6/n‐3 ratio was not <4 in the GZ group, the results showed that it was lower and therefore better in the muscles of steers from the GZ group than in those from the CT group.

The WB shear force of muscles has been found to be higher in pasture‐fed steers than in concentrate‐fed steers (Mitchell, Reed, & Rogers, 1991; Muramoto et al., 2005). In contrast, beef from pasture‐fed steers was more tender than that from indoor‐finished steers at 24 hr *post mortem*, but there was no difference in meat tenderness between the two groups after 7 days of storage (Varela et al., 2004). Beef from grazing cattle tends to be tougher than that from cattle fed concentrates, but in this study, the two feeding regimes did not affect the WB shear force of the muscle samples. In general, marbled beef is more tender than lean beef, because the fat in the muscle improves meat tenderness by reducing the strength of the connective tissue (Savell & Cross, 1988). Pasture‐fed steers (Descalzo et al., 2005; Muramoto et al., 2005), rWCS‐fed steers (Shibata et al., 2016), and grass silage‐fed steers (Warren et al., 2008) have been found to have less intramuscular fat than concentrate‐fed steers. Compared with the CT group, the GZ group had less intramuscular fat but had a fat content at least 20% higher in the LL muscle, so the WB shear force may tend to be low. However, it is difficult to evaluate the tenderness of beef based only on the fat content. In this regard, further histological studies are needed.

## 5 CONCLUSIONS

The results showed that a system for fattening steers combining grazing with feeding rWCS resulted in roughly similar levels of beef productivity to that achieved by feeding a concentrate‐based diet. Grazing steers tended to have a lower BW than that of concentrate‐fed steers. However, the transcript levels of genes involved in muscle growth and the growth performance indices indicated that compensatory growth occurred in steers fed rWCS in a barn after grazing. Although there was no difference in the final BW between the two groups, the grazed steers had a lower dressed weight. Despite the lower intramuscular fat content in the grazed steers, there was no difference in BMS between the two groups. In addition, the intramuscular vitamin content was higher in the grazed steers than in the concentrate‐fed steers.

## References

[asj13176-bib-0001] AOAC . (1990). Official methods of analysis (15th ed.). Arlington, VA: Association of Official Analytical Chemists.

[asj13176-bib-0002] AOCS . (2005). Official methods and recommended practices (5th ed.). Urbana, IL: The American Oil Chemists Society.

[asj13176-bib-0003] Bidner, T. D. , Schupp, A. R. , Mohamad, A. B. , Rumore, N. C. , Montgomery, R. E. , Bagley, C. P. , & McMillin, K. W. (1986). Acceptability of beef from Angus‐Hereford or Angus‐Hereford‐Brahman steers finished on all‐forage or a high‐energy diet. Journal of Animal Science, 62, 381–387.

[asj13176-bib-0004] Bjorklund, E. A. , Heins, B. J. , DiCostanzo, A. , & Chester‐Jones, H. (2014). Growth, carcass characteristics, and profitability of organic versus conventional dairy beef steers. Journal of Dairy Science, 97, 1817–1827. 24472124 10.3168/jds.2013-6983

[asj13176-bib-0005] Bodine, S. C. , Latres, E. , Baumhueter, S. , Lai, V. K. , Nunez, L. , Clarke, B. A. , … Glass, D. J. (2001). Identification of ubiquitin ligases required for skeletal muscle atrophy. Science, 294, 1704–1708. 11679633 10.1126/science.1065874

[asj13176-bib-0006] Calder, P. C. (2004). n‐3 Fatty acids and cardiovascular disease: Evidence explained and mechanisms explored. Clinical Science, 107, 1–11. 15132735 10.1042/CS20040119

[asj13176-bib-0007] Carstens, G. E. , Johnson, D. E. , Ellenburger, M. A. , & Tatum, J. D. (1991). Physical and chemical components of the empty body during compensatory growth in beef steers. Journal of Animal Science, 69, 3251–3264. 1894561 10.2527/1991.6983251x

[asj13176-bib-0008] Department of Health . (1994). Report on health and social subjects No. 46. Nutritional aspects of cardiovascular disease. London, UK: Her Majesty's Stationery Office.

[asj13176-bib-0009] Descalzo, A. M. , Insani, E. M. , Biolatto, A. , Sancho, A. M. , Garcia, P. T. , Pensel, N. A. , & Josifovich, J. A. (2005). Influence of pasture or grain‐based diets supplemented with vitamin E on antioxidant/oxidative balance of Argentine beef. Meat Science, 70, 35–44. 22063278 10.1016/j.meatsci.2004.11.018

[asj13176-bib-0010] Gomes, M. D. , Lecker, S. H. , Jagoe, R. T. , Navon, A. , & Goldberg, A. L. (2001). Atrogin‐1, a muscle‐specific F‐box protein highly expressed during muscle atrophy. Proceedings of the National Academy of Sciences of the United States of America, 98, 14440–14445. 11717410 10.1073/pnas.251541198 PMC64700

[asj13176-bib-0011] Grobet, L. , Martin, L. J. R. , Pirottin, D. , Brouwers, B. , Riquet, J. , Schoeberlein, A. , … Georges, M. (1997). A deletion in the bovine myostatin gene causes the double‐muscled phenotype in cattle. Nature Genetics, 17, 71–74. 9288100 10.1038/ng0997-71

[asj13176-bib-0012] Horton, G. M. J. , & Holmes, W. (1978). Compensatory growth by beef cattle at grassland or on an alfalfa‐based diet. Journal of Animal Science, 46, 297–302.

[asj13176-bib-0013] JMGA . (1988). New beef carcass grading standards. Tokyo, Japan: Japan Meat Grading Association.

[asj13176-bib-0014] Kambadur, R. , Sharma, M. , Smith, T. P. L. , & Bass, J. J. (1997). Mutations in myostatin (GDF8) in double‐muscled Belgian Blue and Piedmontese cattle. Genome Research, 7, 910–915. 9314496 10.1101/gr.7.9.910

[asj13176-bib-0015] Lecker, S. H. , Jagoe, R. T. , Gilbert, A. , Gomes, M. , Baracos, V. , Bailey, J. , … Goldberg, A. L. (2004). Multiple types of skeletal muscle atrophy involve a common program of changes in gene expression. FASEB Journal, 18, 39–51. 14718385 10.1096/fj.03-0610com

[asj13176-bib-0016] MAFF . (2018). Annual report on food, agriculture, and rural areas in Japan. Tokyo, Japan: Ministry of Agriculture, Forestry and Fisheries. (In Japanese).

[asj13176-bib-0017] McPherron, A. C. , Lawler, A. M. , & Lee, S. J. (1997). Regulation of skeletal muscle mass in mice by a new TGF‐β superfamily member. Nature, 387, 83–90. 9139826 10.1038/387083a0

[asj13176-bib-0018] McPherron, A. C. , & Lee, S. J. (1997). Double muscling in cattle due to mutations in the myostatin gene. Proceedings of the National Academy of Sciences of the United States of America, 94, 12457–12461. 9356471 10.1073/pnas.94.23.12457 PMC24998

[asj13176-bib-0019] Mitchell, G. E. , Reed, A. W. , & Rogers, S. A. (1991). Influence of feeding regimen on the sensory qualities and fatty acid contents of beef steaks. Journal of Food Science, 56, 1102–1103.

[asj13176-bib-0020] Montgomery, J. L. , Allen, V. G. , Pond, K. R. , Miller, M. F. , Wester, D. B. , Brown, C. P. , … Fontenot, J. P. (2001). Tasco‐Forage: IV. Influence of a seaweed extract applied to tall fescue pastures on sensory characteristics, shelf‐life, and vitamin E status in feedlot‐finished steers. Journal of Animal Science, 79, 884–894. 11325193 10.2527/2001.794884x

[asj13176-bib-0021] Muramoto, T. , Aikawa, K. , Shibata, M. , & Nakanishi, N. (2002). Effect of restricted feeding of concentrate over the entire fattening period on beef productivity of Japanese Black Steers. Nihon Chikusan Gakkaiho, 73, 57–62. (In Japanese).

[asj13176-bib-0022] Muramoto, T. , Higashiyama, M. , & Kondo, T. (2005). Effect of pasture finishing on beef quality of Japanese Shorthorn Steers. Asian‐Australasian Journal of Animal Sciences, 18, 420–426.

[asj13176-bib-0023] Neill, S. , Unruh, J. A. , Marston, T. T. , Jaeger, J. R. , Hunt, M. C. , & Higgins, J. J. (2008). Effects of implanting and feeding zilpaterol hydrochloride on performance, carcass characteristics, and subprimal beef yields of fed cow. Journal of Animal Science, 87, 704–710. 18820157 10.2527/jas.2008-1254

[asj13176-bib-0024] Pavan, E. , & Duckett, S. K. (2008). Corn oil or corn grain supplementation to steers grazing endophyte‐free tall fescue. I. Effects on in vivo digestibility, performance, and carcass quality. Journal of Animal Science, 86, 3215–3223. 18539823 10.2527/jas.2007-0703

[asj13176-bib-0025] Realini, C. E. , Duckett, S. K. , Hill, N. S. , Hoveland, C. S. , Lyon, B. G. , Sackmann, J. R. , & Gillis, M. H. (2005). Effect of endophyte type on carcass traits, meat quality, and fatty acid composition of beef cattle grazing tall fescue. Journal of Animal Science, 83, 430–439. 15644516 10.2527/2005.832430x

[asj13176-bib-0026] Savell, J. W. , & Cross, H. R. (1988). The role of fat in the palatability of beef, pork, and lamb. In National Research Council (Ed.), Designing foods, Animal product options in the marketplace (pp. 345–355). Washington, DC: National Academy Press.

[asj13176-bib-0027] Shibata, M. , Hikino, Y. , Imanari, M. , Matsumoto, K. , & Yamamoto, N. (2016). Influence of a rice whole‐crop silage diet on growth performance, carcass and meat characteristics, and muscle‐related gene expression in Japanese Black steers. Animal Science Journal, 87, 929–937. 26420580 10.1111/asj.12519

[asj13176-bib-0028] Shibata, M. , Hikino, Y. , & Matsumoto, K. (2019). Influence of feeding a grass hay diet during the early stage of the fattening period on growth performance, carcass characteristics and meat production in Japanese Black steers. Animal Science Journal, 10.1111/asj.13139. [Epub ahead of print]. PMC6590438 30561151

[asj13176-bib-0029] Shibata, M. , Matsumoto, K. , Aikawa, K. , Muramoto, T. , Fujimura, S. , & Kadowaki, M. (2006). Gene expression of myostatin during development and regeneration of skeletal muscle in Japanese Black Cattle. Journal of Animal Science, 84, 2983–2989. 17032792 10.2527/jas.2006-118

[asj13176-bib-0030] Shibata, M. , Matsumoto, K. , Hikino, Y. , Oe, M. , Ojima, K. , Nakajima, I. , … Chikuni, K. (2011). Influence of different feeding systems on the growth performance and muscle development of Japanese Black steers. Meat Science, 89, 451–456. 21641731 10.1016/j.meatsci.2011.05.006

[asj13176-bib-0031] Shibata, M. , Matsumoto, K. , Hikino, Y. , Oe, M. , Ojima, K. , Nakajima, I. , … Yamamoto, N. (2012). Effect of grass hay feeding on meat production, carcass characteristics, and meat quality in japanese black steers. Bulletin of NARO Western Region Agricultural Research Center, 11, 15–25.

[asj13176-bib-0032] Shibata, M. , Matsumoto, K. , Hikino, Y. , & Yamamoto, N. (2014). Effect of indoor concentrate feeding vs. outdoor grazing on the expression of genes involved in muscle growth and nutrient content in Japanese Black steer muscle. Open Journal of Animal Sciences, 4, 297–304.

[asj13176-bib-0033] Shibata, M. , Matsumoto, K. , Oe, M. , Ohnishi‐Kameyama, M. , Ojima, K. , Nakajima, I. , … Chikuni, K. (2009). Differential expression of the skeletal muscle proteome in grazed cattle. Journal of Animal Science, 87, 2700–2708. 19420231 10.2527/jas.2008-1486

[asj13176-bib-0034] Takahira, Y. , Kanaya, C. , Yoshino, E. , Kon, H. , Maruyama, T. , & Kasuya, K. (2011). Effects of feeding whole‐crop rice silage with reduced β‐carotene content on various characteristics of Japanese Black steers. Japan Journal of Grassland Science, 56, 245–252. (In Japanese).

[asj13176-bib-0035] Varela, A. , Oliete, B. , Moreno, T. , Portela, C. , Monserrat, L. , Carballo, J. A. , & Sanchez, L. (2004). Effect of pasture finishing on the meat characteristics and intramuscular fatty acid profile of steers of the Rubia Gallega breed. Meat Science, 67, 515–522. 22061527 10.1016/j.meatsci.2003.12.005

[asj13176-bib-0036] Warren, H. E. , Scollan, N. D. , Enser, M. , Hughes, S. I. , Richardson, R. I. , & Wood, J. D. (2008). Effects of breed and a concentrate or grass silage diet on beef quality in cattle of 3 ages. I: Animal performance, carcass quality and muscle fatty acid composition. Meat Science, 78, 256–269. 22062278 10.1016/j.meatsci.2007.06.008

[asj13176-bib-0037] Wing, S. S. , Haas, A. L. , & Goldberg, A. L. (1995). Increase in ubiquitin protein conjugates concomitant with the increase in proteolysis in rat skeletal muscle during starvation and atrophy denervation. Biochemical Journal, 307, 639–645. 10.1042/bj3070639 PMC1136698 7741691

